# Safety and efficacy of bridging to lung transplantation with antifibrotic drugs in idiopathic pulmonary fibrosis: a case series

**DOI:** 10.1186/s12890-016-0308-z

**Published:** 2016-11-18

**Authors:** Isabelle Delanote, Wim A. Wuyts, Jonas Yserbyt, Eric K. Verbeken, Geert M. Verleden, Robin Vos

**Affiliations:** 1 Department of Respiratory Diseases, Interstitial Lung Disease and Lung Transplant Unit, University Hospitals Leuven, Leuven, Belgium; 2 KULeuven, Department of Clinical and Experimental Medicine, Division of Respiratory Diseases, Laboratory of Respiratory Diseases, Lung Transplantation Unit, KU Leuven, Herestraat 49, B-3000 Leuven, Belgium; 3 KULeuven, Department of Histopathology, Leuven, Belgium

**Keywords:** Antifibrotics, IPF, Lung transplantation, Nintedanib, Pirfenidone, Safety

## Abstract

**Background:**

Following recent approval of pirfenidone and nintedanib for idiopathic pulmonary fibrosis (IPF), questions arise about the use of these antifibrotics in patients awaiting lung transplantation (LTx).

**Methods:**

Safety and efficacy of antifibrotic drugs in IPF patients undergoing LTx were investigated in a single-centre retrospective cohort analysis.

**Results:**

A total of nine patients, receiving antifibrotic therapy for 419 ± 315 days until subsequent LTx, were included. No major side effects were noted. Significant weight loss occurred during antifibrotic treatment (*p* = 0.0062). FVC tended to stabilize after 12 weeks of treatment in most patients. A moderate decline in FVC, TLC and DLCO was noted during the whole pretransplant time period of antifibrotic therapy. Functional exercise capacity and lung allocation score remained unchanged. No post-operative thoracic wound healing problems, nor severe early anastomotic airway complications were attributable to prior antifibrotic treatment. None of the patients developed chronic lung allograft dysfunction after a median follow-up of 19.8 (11.2–26.5) months; and post-transplant survival was 100% after 1 year and 80% after 2 years.

**Conclusions:**

Antifibrotic drugs can probably be safely administered in IPF patients, possibly attenuating disease progression over time, while awaiting LTx.

## Background

Idiopathic pulmonary fibrosis (IPF) is a progressive and lethal disease characterized by chronic, fibrosing interstitial pneumonitis of unknown cause, associated with a histopathologic and/or radiologic pattern of usual interstitial pneumonia (UIP) [1]. The course of the disease is unpredictable. Most patients demonstrate a slow, gradual progression; some patients remain stable; while others have an accelerated decline, sometimes due to repeated exacerbations. Consequently, respiratory failure is the most common cause of death in IPF. Once diagnosed, timely referral to an expert centre is therefore essential to assess eligibility for pharmacological therapy and/or lung transplantation (LTx) [2].

In October 2014, the US Food and Drug Administration (FDA) approved two anti-fibrotic drugs for IPF - pirfenidone and nintedanib - based on the results of large randomized clinical trials (CAPACITY-1, CAPACITY-2 and ASCEND with pirfenidone; TOMORROW, INPULSIS-1 and INPULSIS-2 with nintedanib) demonstrating a reduction in the rate of decline in forced vital capacity (FVC) in mild to moderate IPF [3–5]. Post-hoc analysis also demonstrated a risk reduction for IPF-related mortality with pirfenidone compared to placebo (HR 0.32, 95% CI 0.14–0.76, *p* = 0.006) [6], a same trend which was also observed with nintedanib (HR 0.70; 95% CI 0.46–1.08; *p* = 0.0954) [7].

Despite these positive findings, it should be emphasized that both antifibrotic drugs do not represent a ‘cure’ for IPF, but only aim to attenuate the decline in FVC, at best resulting in temporary disease stabilization. Moreover, side effects (typically nausea, anorexia, malaise, or rash for pirfenidone; and diarrhea for nintedanib) or adverse events (mainly toxic hepatitis) may force some patients to reduce or even stop treatment, which may again accelerate disease progression. Hence, early evaluation and referral for LTx, which presently remains the only definitive treatment option for well-selected IPF patients, is highly recommendable, particularly since IPF patients have the highest waiting list mortality, due to disease progression. The recent introduction of the lung allocation score (LAS) in some countries may nevertheless decrease future waiting list mortality in IPF. Implementation of the LAS indeed has already led to a substantial increase in the proportion of LTx performed for IPF, making it the most common indication for LTx and reducing waiting list time for IPF in these countries [8].

With increasing use of antifibrotics following recent FDA approval, questions arise about their safety in IPF patients undergoing LTx, yet safety data in this specific setting are currently lacking. The antifibrotic properties of pirfenidone result from inhibition of transforming growth factor (TGF)-β expression, thus attenuating myofibroblast differentiation and fibroblast activity [9]. Nintedanib is a tyrosine kinase inhibitor, which blocks receptors for platelet-derived growth factor (PDGF), fibroblast growth factor (FGF), and vascular endothelial growth factor (VEGF), thus inhibiting downstream signaling in (myo-)fibroblasts [10]. Both antifibrotics may hence theoretically impair post-operative wound healing and/or cause bronchial anastomotic complications following LTx. Nintedanib, by inhibition of VEGF and PDGF, may in theory also result in an increased peri-operative bleeding risk. Moreover, it is unclear whether antifibrotic treatment, when effectively achieving disease stabilization for several months, would influence LAS or may even interfere with referral for LTx, given an upper age limit for LTx used in most centres.

In the current study we therefore report on safety and efficacy of pretransplant antifibrotics in IPF patients undergoing LTx. Pretransplant pulmonary function, functional exercise capacity; and immediate and long-term post-operative outcomes, including the early post-operative course, presence of bronchial anastomotic complications, chronic lung allograft dysfunction (CLAD) and survival, were retrospectively assessed.

## Methods

### Study design and population

This is a single-centre, retrospective analysis of IPF patients undergoing LTx in a large volume transplant centre at a tertiary care hospital. The current study was approved by the Leuven University Hospital Ethical Review Board (S51577) and patients gave informed consent. IPF diagnosis was confirmed in by a multidisciplinary board discussion, including an expert chest physician specialized in interstitial lung disease (ILD) (WW), an experienced chest imaging radiologist and a specialized lung-pathologist (EKV). For the current study, we included all IPF patients up to December 2015 who had undergone LTx in our centre whilst being treated with either pirfenidone or nintedanib. There were no IPF patients receiving antifibrotic drugs who died on the waiting list before LTx.

Pirfenidone was initiated between September 2008 and September 2013; and patients were subsequently transplanted between November 2008 and April 2015. Nintedanib was started between August 2010 and January 2012; and patients were transplanted between March 2011 and October 2014. In Belgium, pirfenidone was approved for mild to moderate IPF (FVC >50%predicted (%pred) and Diffusion Capacity (DL_CO_) >35%pred) in December 2012 and nintedanib has been approved for mild to severe IPF (FVC ≥50%pred and DL_CO_ ≥30%pred) since December 2015. Patients in whom antifibrotic therapy was initiated before these respective dates thus received the drugs in the context of clinical trials, thereafter patients received open-label treatment according to reimbursement rules. All IPF patients were evaluated on regular intervals (every 3 to 4 months) at a specialized outpatient ILD consultation by a specialized physician (WW) and nurse, who checked compliance and tolerance of their antifibrotic therapy.

### Data collection

Data were retrospectively collected from the patients’ electronical medical files, including clinical and demographical variables, duration of antifibrotic treatment, laboratory results, anastomotic problems (scored according to MDS classification as previously reported, [11]), evolution of pulmonary function and functional exercise capacity. The estimated annual decline in pulmonary function parameters (FVC, Total Lung Capacity (TLC) and DL_CO_) was calculated based on the difference in pulmonary function parameters between the start of antifibrotic therapy (‘baseline’) and at the time of LTx, adjusted for the number of months therapy was taken (monthly decline) and extrapolated to 1 year (monthly decline x12). The same approach was used regarding the decrease in six minute walking test (6MWT) between start of therapy and LTx. LAS was retrospectively assessed at start of antifibrotic therapy, at LTx listing (data summarized in Table 1) and at LTx. However, we used LAS at start of antifibrotic therapy for further statistical analyses regarding pre-LTx evolution of LAS, because most patients were initiated on antifibrotics before LTx listing.

**Table 1:** Recipient and donor demographics of the IPF treatment group and historical control group

ID	Recipient Gender (M/F)	Recipient Age (Years)	Anti-fibrotic Drug	Cardio-Pulmonary Rehabilitation	Time on Therapy (Days)	FVC at start (%pred)	TLC at start (%pred)	DL_CO_ at start (%pred)	6MWT at start (m)	Time on WL (Days)	LAS at listing	Type of LTx (S/SS)	Donor Gender (M/F)	Donor Age (Years)	Type of Donor	CMV Donor/Recipient
1	F	62	PFD	no	735	91	72	46	529	762	32	SS	F	17	DBD	D+/R+
2	M	61	PFD	CPR	545	71	61	37	379	179	31	SS	F	67	DCD cat III	D-/R-
3	M	51	PFD	CPR	387	88	80	47	552	29	35	SS	M	37	DBD	D+/R-
4	M	63	PFD	CPR	539	52	45	32	384	51	30	SS	M	55	DBD	D-/R+
5	M	55	PFD	CPR	188	56	52	35	631	25	29	SS	M	23	DBD	D+/R-
6	M	64	PFD	no	115	62	48	39	503	163	33	SS	M	35	DCD cat V	D+/R+
7	M	64	PFD	no	65	79	56	28	267	419	37	S	F	42	DBD	D+/R-
8	M	65	NIN	no	1003	80	69	58	598	155	31	SS	M	62	DBD	D+/R-
9	M	56	NIN	no	194	58	56	29	275	74	32	SS	M	39	DCD cat III	D-/R-
	Mean or Median	60.1 ± 4.9			419 ± 315	70.8 ± 14.5	59.9 ± 11.7	39.0 ± 9.8	457.6 ± 135.6	155 (40–299)	32.2 ± 2.5			43.6 ± 17.1		
1	M	57	/	no	/	/	/	/	/	279	33	SS	F	48	DCD cat III	D-/R+
2	M	62	/	no	/	/	/	/	/	153	35	SS	F	66	DBD	D+/R+
3	M	55	/	no	/	/	/	/	/	17	34	SS	M	74	DBD	D+/R+
4	M	59	/	CPR	/	/	/	/	/	274	32	SS	M	22	DCD cat III	D-/R+
5	M	59	/	CPR	/	/	/	/	/	253	29	SS	M	57	DBD	D-/R-
6	M	65	/	CPR	/	/	/	/	/	112	26	S	M	37	DBD	D+/R+
	Mean or Median	59.5 ± 3.6^a^								203 (88–275)^a^	31.5 ± 3.4^a^			50.7 ± 19.^a^		
ID	Ischemic Time 1^th^/2^nd^ Lung (min)	Immuno-suppressive Regimen	Time to Extubation (Hours)	PGD at 72 h	Time on ICU (Days)	Time in Hospital (Days)	AR or LB Episodes (Number)	Most Severe AR or LB (Grade)	Respiratory infection before Discharge (Presence = 1)	Respiratory Pathogen before Discharge	Anastomotic Complications (Details in Text)					
1	187/320	rATG/FK/MMF/CS	34	0	6	16	0	0	0	/	0					
2	432/580	rATG/FK/MMF/CS	20	3	7	20	1	1	1	*E. coli, S. viridans*	M2aD0aS0 (POD 30)					
3	498/694	No ATG, FK/MMF/CS	37	0	4	16	2	2	1	*A. baumanii, E. coli, S. aureus*	M3bD2cS2f (POD 204)					
4	417/631	rATG/FK/MMF/CS	65	1	13	26	1	1	0	/	M2aD0aS0 (POD 30)					
5	366/515	rATG/FK/MMF/CS	41	2	6	17	0	0	1	*C. freundii*	0					
6	385/582	rATG/FK/MMF/CS	37	1	4	16	3	2	0	/	0					
7	341	rATG/CsA/AZA/CS	33	2	3	21	0	0	1	*H. influenza, MRSA*	0					
8	180/356	rATG/FK/MMF/CS	178	3	10	28	1	3	1	*H. influenza, S. pneumoniae*	M1aD0aS0 (POD 90)					
9	239/356	rATG/FK/MMF/CS	38	2	5	23	1	2	0	/	0					
	338 ± 113/504 ± 142		37 (33.5–53)	2 (0.5–2.5)	6.4 ± 3.2	20.3 ± 4.6	1 (0–1.5)	1 (0–2)								
1	404/626	rATG/FK/MMF/CS	72	0	7	19	1	2	0	/	0					
2	220/412	rATG/FK/MMF/CS	16	1	7	25	2	3	1	*S. aureus*	M3bD0aS0 (POD 30)					
3	288/431	rATG/FK/MMF/CS	432	3	60	253	1	1	1	*P. aeruginosa, K. pneumoniae,*	M3bD2bS0 (POD 40)					
4	276/423	rATG/FK/MMF/CS	48	2	15	32	1	1	1	*E. faecalis*	M2aD0aS0 (POD 14)					
5	209/439	rATG/CsA/AZA/CS	48	2	23	32	0	0	1	*K. oxytoca*	M3aD0S0 (POD 17)					
6	186	rATG/CsA/AZA/CS	24	1	8	20	2	1	0	/	0					
	264 ± 79^a^/466 ± 90^a^		48 (22–162)^a^	1.5 (0.8–2.3)^a^	20.0 ± 20.6^b^	63.5 ± 93^a^	1 (0.75–2.0)^a^	1 (0.75–2.25)^a^								
ID	Time of Follow-up(Months)	Status (Dead = 1)	Last FVC Post-LTx (%pred)	Last FEV_1_ Post-LTx (%pred)	Last FEV_1_/FVC Post-LTx (%pred)											
1	7.7	0	145	147	86											
2	8.6	0	106	90	66											
3	16.9	0	70	68	77											
4	21.9	0	90	67	58											
5	25.8	0	93	93	80											
6	27.1	0	123	126	79											
7	19.8	1	110	107	76											
8	13.8	0	119	115	75											
9	56.3	0	132	141	84											
	19.8 (11.2–26.5)		109 ± 23.1	106 ± 29.1	75.7 ± 8.8											
1	14.9	0	117	76	66											
2	23.5	0	104	99	76											
3	37.0	0	67	61	73											
4	38.6	0	107	92	67											
5	52.4	0	104	58	43											
6	12.2	1	79	82	81											
	30.3 (14.2–42.1)		96.3 ± 19.1	78.0 ± 16.4	67.7 ± 13.3											

Data are expressed as mean ± SD, median (interquartile range) or as total values where appropriate

*Abbreviations*: *6MWT* 6 min walking test, *AR* Acute (cellular) Rejection, *AZA* azathioprine, *cat* category, *CMV* Cytomegalovirus, *CPR* Cardio-Pulmonary Rehabilitation, *CS* corticosteroids, *CsA* cyclosporine A, *D* donor, *DBD* donation after brain death, *DCD* donation after cardiac death, *DLCO* diffusion capacity, *F* Female, *FK* tacrolimus, *FVC* Forced Vital Capacity, *ICU* Intensive Care Unit, *ID* identification, *LAS* lung allocation score, *LB* lymphocytic bronchiolitis, *LTx* lung transplantation, *M* male, *MDS* severity of anastomotic complication according to MDS classification, *MMF* mycophenolate mofetil, *NIN* nintedanib, *PFD* pirfenidone, *PGD* primary graft dysfunction, *R* recipient, *rATG* rabbit Anti-Thymocyte Globulin, *S* single, *SS* sequential single, *TLC* Total Lung Capacity, *WL* waiting list

^a^:*p* > 0.05 (not statistically significant compared to treated group), ^b^:*p* = 0.021 compared to treated group

### Historical controls

We additionally identified a comparable group of historical controls (*n* = 6), which consisted of IPF patients who did not receive antifibrotic therapy before LTx, but were transplanted in the same era (7/2010 to 9/2014), had comparable age and lung function at the time of LTx compared to the treatment group: FVC 57.0 (43.0–69.8) %pred (*p* = 0.69 vs. treatment), TLC 53.0 (42.5–71.0) %pred (*p* = 1.0 vs. treatment) and DL_CO_ 21.5 (17.0–29.2) %pred (*p* = 0.11 vs. treatment). Given the small number of available patients, it was impossible to match both groups any further regarding concurrent emphysema (but TLC and DL_CO_ were comparable between both groups, thus excluding major differences due to emphysema), pulmonary hypertension (was not routinely assessed in non-treated IPF patients, no comparison possible with treated group who were all screened at start of antifibrotic therapy) or cardiovascular disease (but major cardiovascular disease is generally an exclusion-criterion to proceed to LTx in any patient). Reasons for not starting antifibrotic therapy in these matched historical controls were: absence of consent (*n* = 3), DL_CO_ too low for study-inclusion (*n* = 2) and pending approval by the health care authorities whilst awaiting LTx (*n* = 1). These historical controls were only used as comparator for the IPF group treated with antifibrotics regarding the annual pre-transplant decline in pulmonary function; and some mportant early post-transplant outcome parameters, including rates of PGD, infection, rejection and anastomotic complications. These historical patients were not the main aim of this study, which focusses on reporting safety and efficacy of antifibrotics in IPF patients undergoing LTx.

### Statistical analysis

All analyses were performed using Graphpad Prism 5a software (San Diego, USA). Results are expressed as mean (± standard deviation) or median (interquartile range) where appropriate. Group means were compared using paired or unpaired *t*-test; Mann-Whitney test or Wilcoxon signed rank test for normally or not-normally distributed variables, respectively. All reported *p*-values are two-tailed and *p* < 0.05 was considered significant.

## Results

### Patients’ characteristics

A total of 9 IPF patients were treated with antifibrotics and subsequently underwent LTx: pirfenidone *n* = 7 (*n* = 2 study vs. *n* = 5 open-label treatment), nintedanib *n* = 2 (both in study). All patients, but one, underwent bilateral LTx and all, but one, were male. Age at LTx was 60.1 ± 4.9 years. Five patients were on continuous oxygen therapy (4 (3.5–4.0) Liters/min) before LTx, while 4 were not (Table 1). Antifibrotic therapy had been initiated 362 (152–578) days before listing for LTx in 6/9 patients, whereas in 3/9 patients antifibrotics were started 48 (27–354) days after transplant listing. In all 9 cases antifibrotic therapy was continued until the day of transplant procedure. Total duration of antifibrotic therapy until LTx was 419 ± 315 days, or 13.8 ± 10.3 months. All patients received the full, recommended dose (i.e. 801 mg tid for pirfenidone and 150 mg bid for nintedanib).

Nausea was reported as main side-effect of antifibrotic therapy in 9/9 patients; and 7/9 patients lost weight during treatment (*n* = 6 pirfenidone, *n* = 1 nintedanib), in one patient (on pirfenidone) weight remained stable and one patient (on nintedanib) gained 1 kg. Overall, body mass index (BMI) decreased from 27.3 ± 3.2 kg/m^2^ to 25.8 ± 3.3 kg/m^2^ (*p* = 0.0063) during antifibrotic treatment, with an absolute weight loss of 329 ± 360 g per month of treatment (*p* = 0.0062). None of the patients developed toxic hepatitis, nor discontinued their therapy due to other severe side-effects or adverse events. No acute IPF exacerbations occurred in any of the patients during antifibrotic treatment.

### Evolution of pretransplant pulmonary function, functional exercise capacity, pulmonary hypertension, renal function and LAS

**Fig. 1:**
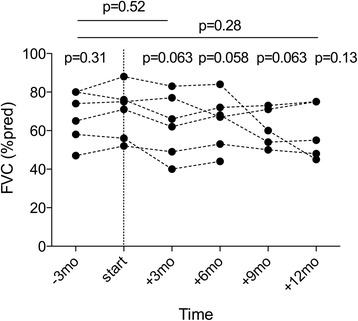
Forced Vital Capacity in IPF patients with at least 6 months antifibrotic therapy before transplantation. Forced Vital Capacity (FVC) (%predicted) is given at the start of antifibrotic therapy (start), 3 months before and respectively 3, 6, 9 and 12 months (mo) after start. Dotted lines connect values in patients (*n* = 6/9) with consecutive measurements at different time points; *p*-values (Wilcoxon signed rank test) above each time point are given compared to start; or compared another time point (time-frame indicated by full line)

**Fig. 2:**
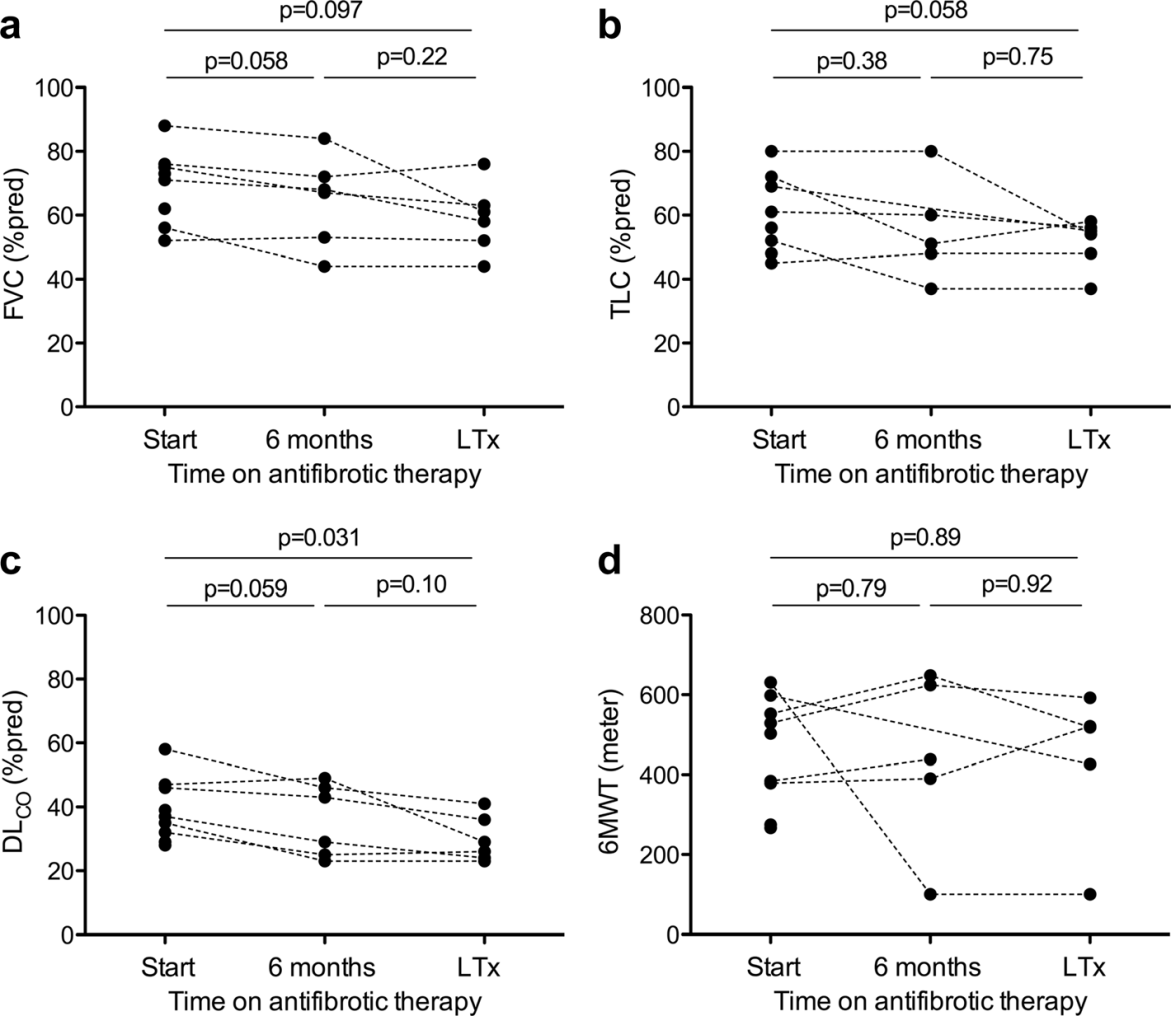
Pretransplant evolution of pulmonary function and functional exercise capacity following treatment with antifibrotic drugs. Forced Vital Capacity (FVC) (**a**), Total Lung Capacity (TLC) (**b**), Diffusion capacity (DL_CO_) (**c**) (all in (%predicted) and 6 min walk test (6MWT, meter) (**d**) at start of antifibrotic therapy (start) and at the moment of lung transplantation (LTx) in the included IPF patients. Dotted lines connect values in patients (*n* = 6/9) with a consecutive measurement at six months and just before transplantation; *p*-values (Wilcoxon signed rank test) are given for patients that had consecutive measurements

Spirometry was performed at the start of antifibrotic treatment (‘baseline’) and during subsequent follow-up. Consecutive spirometry after six months of antifibrotic therapy was only available in 6/9 patients, as 3 patients (*n* = 2 pirfenidone, *n* = 1 nintedanib) underwent LTx within 6 months after initiating therapy (Fig. 1). In these 6/9 patients (*n* = 5 pirfenidone, *n* = 1 nintedanib), the absolute decline in FVC *after 12 weeks* of treatment compared to baseline was −7.0%pred (−1.8 to −11.5), with 4/6 (66.6%) patients having <10% decline in FVC %pred; and only 2/6 (33.3%) patients demonstrating a ≥10% decline in FVC %pred (*p* = 0.063 vs. start). Nevertheless, an overall absolute decrease in FVC, TLC and DL_CO_ during the *whole* pretransplant antifibrotic treatment period (i.e. 419 ± 315 days or 59 ± 44 weeks) was observed in these 6/9 patients (Fig. 2).

The calculated *annual* decline during treatment for all included patients was: FVC 322.0 (148.3–1074.0) mL or 6.6 (0–23.8) %pred, TLC 360.0 (157.5–1818.0) mL or 6.0 (2.0–25.7) %pred; and DL_CO_ 0.77 (0.40–1.96) mmol/min/Kpa or 7.5 (4.7–18.6) %pred. Interestingly, the measured annual rate of decline in the matched historical controls (without antifibrotic therapy) during the year preceding LTx appeared to be somewhat more severe compared to the group with antifibrotics, although no significant differences were seen: FVC 460.0 (215.0–732.5) mL or 13.0 (4.8–18.0) %pred (*p* = 0.69); TLC 945.0 mL (362.5–1490) or 10.0%pred (2.0–20.0) (*p* = 1.0); and DL_CO_ 1.26 (0.38–2.09) mmol/min/Kpa or 14.0 (4.0–24.8) %pred (*p* = 0.94).

6MWT was performed before the start of antifibrotic therapy and consecutive 6MWT was available in 5/9 patients (all on pirfenidone), of whom 3/5 were enrolled in a pretransplant cardio-pulmonary rehabilitation (CPR) program upon transplant listing and 2/5 were not. 6MWT overall increased with 54 (−260.0–95.5) m *after 12 weeks* of treatment compared to baseline (*p* = 0.62), with an improved in 4/5 patients of 74.5 (21.8–95.8) m, while one patient demonstrated a decline of 531 m (patient n°5 in Table 1, no CPR, concomitant decline in FVC of 12%pred during these 12 weeks of treatment). During the *whole* pretransplant time period of antifibrotic treatment (59 ± 44 weeks), 6MWT did not significantly change compared to baseline (*p* = 0.89): 6MWT improved compared to baseline in 2/5 patients (+63 m (no CPR) and +142 m (with CPR), respectively), while 6MWT deteriorated in 3/5 patients (one no CPR, two with CPR), in whom there was an absolute decline of −172 (34–531) meters or a *monthly* decline of −5.2 (2.7–85.7) meters during treatment (Fig. 2). In the historical controls, unfortunately, 6MWD was only available upon listing for LTx, thus no consecutive 6MWT were available for further comparison.

Transthoracic echocardiography performed before start of antifibrotic therapy (pulmonary arterial pressure (PAP) 31.1 ± 7.4 mmHg) and consecutive echocardiography was available in 4/9 patients, in whom PAP tended to increase during antifibrotic treatment (PAP +9.5 (2.0–15.5) mmHg: *p* = 0.090). Renal function remained stable during antifibrotic treatment: serum creatinine was 0.96 ± 0.14 mg/dL at start versus 0.95 ± 0.17 mg/dL at LTx (*p* = 0.97), estimated glomerular filtration rate was 83 ± 13 mL/min/1.73 m^2^ at start versus 82 ± 14 mL/min/1.73 m^2^ at LTx (*p* = 0.83). No hepatic dysfunction was observed in any patient during treatment. LAS did not significantly change during antifibrotic treatment: 32.2 ± 2.5 at start of therapy versus 32.3 ± 1.0 at LTx (*p* = 0.13).

### Post-transplant outcomes

Patients receiving antifibrotics were listed for 155 (40–299) days before subsequent LTx. Transplant procedures were overall uneventful and only one patient (who had received pirfenidone, had the highest pretransplant PAP of 48 mmHg and underwent single sided LTx) required peri-operative support with veno-arterial extracorporeal membrane oxygenation. There were no bleeding problems (i.e. no need for re-thoracotomy for hemothorax, no additional transfusion of blood products for blood loss) in any patient, including those on nintedanib. Overall, patients were extubated after 37.0 (33.5–53.0) hours of ventilation, discharged from the intensive care unit after 6.4 ± 3.2 days and discharged home after a hospital stay of 20.3 ± 4.6 days. There were no problems with post-operative thoracic wound healing or dehiscence in any patient. All patients, but one, received post-operative induction therapy with anti-thymocyte globulin for 3 days; and post-operative immunosuppressive regimen consisted of tacrolimus, mycophenolate mofetil and steroids in all patients, except one (transplanted in 2008) who received cyclosporine, azathioprine and steroids (our standard regimen before 2010). No major side effects due to possible drug-interactions with prior antifibrotics were seen in the first days post-LTx.

A total of 4/9 patients were included in a clinical trial immediately following LTx: 2 in a therapeutic trial with azithromycin (AZI003, NCT01915082), 1 in an ex-vivo normothermic machine perfusion trial (EXPANDLung, NCT01963780) and 1 in a Diaphragm Pacing trial (NCT02411383), which may obviously influence early and/or late outcomes (including post-transplant evolution of pulmonary function, anastomotic airway complications, primary graft dysfunction (PGD), rejection, infection, CLAD) in these transplant recipients compared to those not included in a trial or historical controls. Overall, incidence of PGD (PGD ≥ 2 in 5/9 patients), early post-operative infection (5/9 patients) and acute cellular rejection (4/9 patients) or lymphocytic bronchiolitis (4/9) during the first 6 months were comparable to findings in the historical controls (all *p* > 0.5) (Table 1).

Anastomotic airway complications were present in 4/9 patients: in two patients (prior pirfenidone) mild anastomotic necrosis without dehiscence or airway narrowing was noted upon discharge after LTx (post-operative day (POD) 30; MDS classification M2aD0aS0 for right-sided anastomosis and M0aD0aS0 for left-sided anastomosis in both patients), with spontaneous and uncomplicated resolution thereafter. In a third patient (initially no anastomotic complications, prior nintedanib), there was mild protrusion of cartilage on POD 90 (M0aD0aS0 for right-sided anastomosis and M1aD0aS0 for left-sided anastomosis), with spontaneous and uncomplicated resolution thereafter. In the fourth patient (initially no anastomotic complications, prior pirfenidone), following infection with *Aspergillus fumigatus* at POD 186, late-onset (POD 204) anastomotic necrosis occurred with bronchial narrowing and extensive dehiscence (M0aD0aS0 for right-sided anastomosis and M3bD2cS2f for left-sided anastomosis). Despite antifungal treatment, he developed severe symptomatic anastomotic stenosis, which finally required surgical sleeve-resection and reconstruction of the left main bronchus on POD 410. Thereafter, no other problems occurred and the patient currently has a stable pulmonary function at POD 525. The observed anastomotic airway complications, however, did not appear to be more severe or prevalent compared to previously reported data from our centre [11] or to the historical controls, of whom 4/6 controls had early anastomotic airway complications (ranging from M2aD0aS0 to M3bD2bS0; Table 1).

Overall, long-term outcome in our cohort was good: after a median follow-up of 19.8 (11.2–26.5) months, currently all patients have a stable pulmonary function (Table 1) and none of the patients has developed CLAD. One patient (who underwent single sided LTx), unfortunately, has died because of non-squamous large cell lung carcinoma of his native IPF lung on POD 615, all other patients are alive and ambulatory at present. Overall survival was 100% after 1 year and 80% after 2 years, respectively.

## Discussion

Little is known about safety of antifibrotic therapy with pirfenidone or nintedanib in patients undergoing LTx. Actually only 11 IPF patients receiving pirfenidone; and none receiving nintedanib, included in the large randomized trials with these drugs (comprising a total of 2832 study-subjects) were reported as having been transplanted during antifibrotic treatment, yet detailed outcome data for these patients are lacking [3–7]. Only 1 case report has currently been published on pretransplant pharmacological bridging with pirfenidone, allowing stabilization of respiratory function and subsequent single sided LTx in IPF. Anastomic airway complications, however, were not reported in this case [12]. Next to this, there have been two abstracts reporting on this topic, which did not yet result in peer-reviewed papers, but in which, apparently, pirfenidone therapy was not linked to adverse post-transplant events, however follow-up was limited and detailed outcome data missing [13, 14]. In the current case series, we therefore report on pre-operative evolution and post-transplant outcomes of 9 IPF patients, treated with either pirfenidone or nintedanib for a mean of 13.4 months until subsequent LTx and with a median post-transplant follow-up of 19.8 months.

According to the same definitions used in larger IPF trials [6, 15], we noted relative stabilization (i.e. < 10% change) of FVC during the first 12 weeks of antifibrotic treatment. Importantly, this early stabilization, or perhaps better attenuated rate of decline, in FVC may by no means be a reason to deny subsequent LTx to eligible patients, because further decline in FVC, lung volumina and DL_CO_ is to be expected despite antifibrotic treatment, as was obvious from our results. The estimated annual decline in FVC during treatment in our cohort, however, would be around 6.6%pred, which corroborates recent findings that both pirfenidone and nintedanib reduce the proportion of patients with a ≥10% decline in FVC %pred after 1 year of treatment [5, 6]. As they may attenuate disease progression, these antifibrotics may thus allow for valuable added time on the LTx waiting list. Next to FVC, 6MWT has also been shown to be a valid outcome measure, both in IPF, in whom the clinically important difference in 6MWT distance is reported to be 24–45 m [3–5] and in whom 6MWT is associated with changes in pulmonary function and quality-of-life [16]; and in patients awaiting LTx, in whom it is associated with post-transplant survival [17]. A reduction of the decline in 6MWT was also observed in treated patients compared to placebo in pooled analyses of IPF trials [3–5], which may partly explain why 6MWT overall remained relatively stable during treatment in our cohort, next to the obvious beneficial effects of cardio-pulmonary rehabilitation is some patients. Although the LAS is actually not used in Belgium for prioritizing organ allocation, the calculated LAS (which includes FVC and 6MWT among other parameters) did not significantly change during pretransplant antifibrotic treatment in our cohort. An average LAS of 32 at the time of LTx in our study may seem fairly low for IPF patients, yet LAS was quite comparable between our treated patients and historical controls; and was in the same range (median of ±35) as previously described for IPF patients at LTx listing [18]. We therefore believe that our cohort indeed reflects the general population of IPF patients transplanted during the past 5–10 years. However, in the last few years, as seen in the US, an increase in LAS is also noted in our centre, with more sicker patients (LAS > 40) being listed for LTx [19].

No serious side effects were noted during antifibrotic therapy. However, significant weight loss occurred, which is most likely due to drug-induced anorexia or possibly due to respiratory cachexia in end-stage lung disease. Post-operatively, no problems with bleeding or thoracic wound healing were observed. One patient, treated with nintedanib; and three patients who had received pirfenidone developed, mostly mild and uneventful, anastomic airway complications. Intervention for anastomotic stenosis was needed for one case, which only occurred late-onset after prior fungal infection. Overall, it is unlikely that any of these anastomotic problems were directly related to prior antifibrotic treatment given the time of onset/clinical context of anastomotic complications, comparable anastomotic problems in the historical controls; and rather short half-life of both drugs (for pirfenidone 3 h, for nintedanib 9.5 h) [20, 21]. The short half-life of both antifibrotic drugs is important, as drug-interactions with calcineurin inhibitors, by altered hepatic (CYP3A4) metabolisation leading to changes in tacrolimus/cyclosporine trough levels, are a feared iatrogenic adverse event in LTx. However, hepatic metabolism of pirfenidone primarily occurs through the CYP1A2 enzyme; whereas nintedanib is mainly a substrate of P-glycoprotein (P-gp) and only weakly interferes with CYP3A4. This probably also explains why no major side effects due to drug-interactions with peri-operatively used drugs were noted in our cohort. Finally, long-term outcomes regarding pulmonary function and overall survival were overall good in our current case series, suggesting that antifibrotic agents can probably be safely given without deleterious effects on peri-operative or medium-term outcomes.

Possible limitations of the current study, of course, are its retrospective design, the small number of included patients; and historical controls as comparator for some outcomes, which of course limits interpretations regarding antifibrotic drug efficacy and safety. Also, disease severity ranged from mild to severe IPF, which may bias the observed effects of pretransplant antifibrotic therapy; and post-transplant evolution, including pulmonary function, may be biased by inclusion of some patients in various randomized clinical trials. Larger, preferably prospective, case-series are therefore undeniably needed to confirm our findings, especially for nintedanib additional safety data are needed before firmer conclusions can be made regarding its safety.

## Conclusion

In summary, we conclude that antifibrotic drugs are probably safe in IPF patients undergoing LTx. By attenuating disease progression while awaiting LTx, these antifibrotics may perhaps further help to reduce the number of IPF patients dying on the waiting list.

## Abbreviations

6MWT: Six minute walking test
BMI: Body mass index
CLAD: Chronic lung allograft dysfunction
CPR: Cardio-pulmonary rehabilitation
DLCO: Diffusion capacity
FDA: Food and Drug Administration
FGF: Fibroblast growth factor
FVC: Forced vital capacity
ILD: Interstitial lung disease
IPF: Idiopathic pulmonary fibrosis
LAS: Lung allocation score
LTx: Lung transplantation
MDS: Macroscopic Diameter Sutures (MDS Classification)
PDGF: Platelet-derived growth factor
PGD: Primary graft dysfunction
POD: Post-operative day
TGF- β: Transforming growth factor –beta
TLC: Total lung capacity
UIP: Usual interstitial pneumonia
VEGF: Vascular endothelial growth factor
